# Fe (III)-Mediated Antioxidant Response of the Acidotolerant Microalga *Coccomyxa onubensis*

**DOI:** 10.3390/antiox12030610

**Published:** 2023-03-01

**Authors:** María Robles, Rafael Torronteras, Carol Ostojic, Cinta Oria, María Cuaresma, Inés Garbayo, Francisco Navarro, Carlos Vílchez

**Affiliations:** 1 Algal Biotechnology, CIDERTA-RENSMA, Faculty of Experimental Sciences, University of Huelva, 21007 Huelva, Spain; 2 Biology and Environmental Analysis, RENSMA, Faculty of Experimental Sciences, University of Huelva, 21007 Huelva, Spain; 3 Cell Alterations by Exogenous Agents, RENSMA, Faculty of Experimental Sciences, University of Huelva, 21007 Huelva, Spain

**Keywords:** antioxidants, oxidative stress, Fe, reactive oxygen species, photosynthesis

## Abstract

*Coccomyxa onubensis* (*C. onubensis*) is an acidotolerant microalga isolated from Tinto River (Huelva), which contains high levels of metal cations in solution, mainly Fe (II) and (III), and Cu (II). Fe is more bioavailable at low pH, mainly because Fe (II) and Fe (III) are far more soluble, especially Fe (III). For this reason, this study aims to evaluate both physiological and biochemical responses of *C. onubensis* when subjected to Fe (III)-induced stress. Changes in growth, photosynthetic viability and antioxidant responses to the induced oxidative stress were determined. The results obtained suggest that the addition of moderate Fe (III) levels to *C. onubensis* cultures results in improved growth and photosynthetic viability. Increases in the intracellular levels of the enzyme superoxide dismutase (SOD) and flavonoids, used as antioxidant response biomarkers, a point at Fe (III)-mediated oxidative stress induction. The apparent decrease in the content of other phenolic molecules and polyunsaturated fatty acids might be understood as a sign of antioxidant molecules' involvement in reactive oxygen species (ROS) scavenging. In conclusion, a noticeable antioxidant capacity displayed by *C. onubensis* allows the use of moderate Fe (III) levels to trigger the accumulation of valuable antioxidant molecules, allowing the production of cell extracts with potential anti-inflammatory activity.

## 1. Introduction

Microalgae in nature are usually subjected to abiotic stress, which may be due to changes in the salinity of the environment, temperature variation, ultraviolet radiation, lack of nutrients or presence of metal cations, among other factors. Under these extreme conditions, microalgae produce certain metabolites to maintain homeostasis by minimizing the impact of stress. Some metabolites produced to counteract the stress impact have commercial value [1], including terpenoids, phenolic compounds, polyunsaturated fatty acids (PUFAs) and even polysaccharides, among others, with antioxidant, antimicrobial and anti-inflammatory bioactivity. PUFAs, particularly long-chain omega-3 fatty acids, are highly susceptible to oxidation, as their double bonds are targeted by reactive oxygen species (ROS), producing lipoperoxides. Carotenoids are antioxidant molecules, and microalgae increase their intracellular concentration to eliminate excess free radicals derived from oxidative stress [2]. Polyphenols quench free radicals, and this chemical capacity has evidenced their efficiency in cellular protection against oxidative stress. Flavonoids display a range of cell functions, including acting as redox transfer moieties and UV radiation dissipation. The above-referred biomolecules share common applications based on their proven antioxidant capacity: a number of microalgal PUFAs, carotenoids and phenolic compounds have been reported to display biological activities valuable to human health, including anticancer, antioxidant and anti-inflammatory capacities [3,4]. Therefore, incubation conditions for microalgae cultures leading to the accumulation of these molecules would be highly useful to efficiently design biotechnological processes to obtain extracts with potential bioactivity.

*Coccomyxa onubensis* (*C. onubensis*) is an acidotolerant eukaryotic microalga [5], isolated from acid mine drainages of the pyritic belt in the province of Huelva, Spain, characterized by the presence of cations in solution, of which Fe (II) and (III) and Cu (II) have a relevant quantitative presence [6]. It has been shown that the extremely acidic conditions of the Tinto river basin are not just the product of 5000 years of mining activity in the area but the consequence of an active underground bioreactor that obtains its energy from the sulfur minerals of the Iberian Pyrite Belt [6]. The bio-oxidation of pyrite produces a sulfate-enriched acidic solution (pH between 0.8 and 3) that prevents ferric iron precipitation [7], contrary to what occurs in neutral conditions. Under these extremely acidic conditions, ferric iron concentrations as high as 30 g·L^−1^ may be recorded [8]. *C. onubensis* is an example of a photosynthetic microorganism adapted to the extremely acidic pH waters and high levels of solved metals of that environment. Under certain cultivation conditions, *C. onubensis* stands out for accumulating antioxidant molecules such as linoleic and linolenic acid and lutein [9], which have been reported to display anti-inflammatory and antimicrobial activities [10].

Fe is essential in microalgae for redox processes, being an essential component of S-Fe clusters of proteins involved in electron transport reactions; on the other hand, Fe also triggers oxidative stress addressing the cell metabolism to synthesize antioxidant molecules such as polyphenols or carotenoids and to express the synthesis of antioxidant enzymes. Fe is toxic at high concentrations as it can interact with active centers of enzymes or interfere with redox processes [11]. Its intracellular presence, in both forms Fe (II) and Fe (III), produces the well-known Fenton reaction [12], giving rise to ROS that would act in molecular signaling to activate the expression of genes responsible for antioxidant molecules and enzyme production [13].

**Figure 1:**
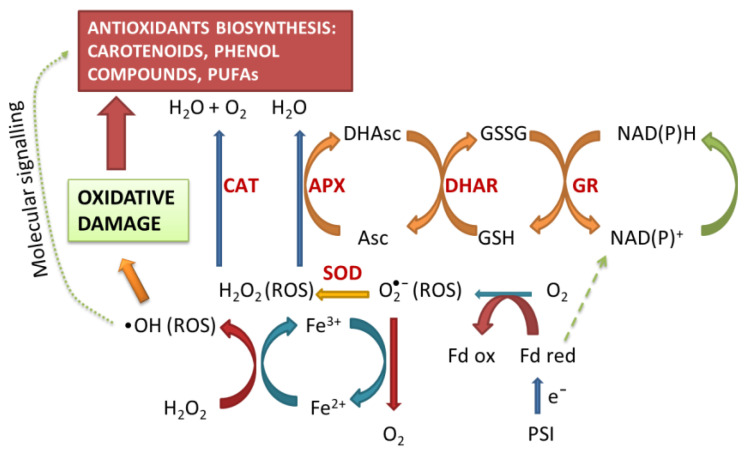
Reactive oxygen species (ROS) formation and antioxidant enzymatic system in microalgae. GSSG: oxidized glutathione; GSH: reduced glutathione; NADP (+,H): nicotinamide adenine dinucleotide phosphate (oxidized, reduced); Asc: reduced ascorbate; DHAsc: dehydroascorbate; CAT: catalase; SOD: superoxide dismutase; APX: ascorbate peroxidase; DHAR: dehydroascorbate reductase; GR: glutathione reductase; Fd red: reduced ferredoxin, with electrons from PSI; Fd ox: oxidized ferredoxin.

As shown in Figure 1, the intracellular presence of Fe (II) or Fe (III) results in the production of compounds that are detrimental to microalgal viability, ROS. Nevertheless, ROS are unavoidably produced in the cell, and thus they may have an important function as signaling molecules [14]. An imbalance of ROS causes strong oxidative damage as they react with proteins, lipids, and nucleic acids to produce their oxidation. ROS originate from electron transport processes, the photosynthetic electron transport chain being a major electron source. Superoxide radical (O_2_^•−^), besides singlet oxygen, is usually the first ROS to be formed. An intensive activity of the photosynthetic electron transport chain favors molecular oxygen (O_2_) to accept a single electron [15]. When O_2_^•−^ is reduced by a second electron, hydrogen peroxide (H_2_O_2_) is produced. O_2_^•−^ and H_2_O_2_ undergo transformation into the more reactive and toxic form, ^•^OH [16]. This oxygen species causes oxidative damage in cell membranes as ^•^OH groups attack the double bonds of phospholipids giving rise to epoxy groups, which promotes stronger membrane lipids hydrocarbon chains interaction, thus addressing membrane fluidity losses. Moreover, ^•^OH can also attack DNA nitrogen bases, giving rise to mutations that seriously impair cell life [15].

At high concentrations, Fe is one of the metals heading the molecular signaling that induces the expression of genes encoding the following antioxidant enzymes: ascorbate peroxidase, superoxide dismutase (SOD), dehydroascorbate reductase and catalase [17,18]. SOD is a metalloprotein responsible for catalyzing the transformation of O_2_^•−^ into H_2_O_2_. In prokaryotes, Mn-SOD and Fe-SOD can be found, while in eukaryotes, Cu/Zn-SOD stands out [17,19].

We hypothesize that modulate stress triggered by Fe (II) and/or Fe (III) in acidophilic or acid-tolerant microalgal cultures, adapted to cope with oxidative conditions, might address increased productivity of antioxidant molecules (flavonoids, other phenolic compounds, PUFAs and/or carotenoids). The photochemical activity of stressed cells would be expected to be less negatively affected than that of common microalgae under similar conditions, according to previous reports referring to the strong antioxidant enzyme response of *C. onubensis* subjected to oxidative stress [9]. Thus, this study analyzes the microalgal growth and antioxidant response of *C. onubensis* to Fe-mediated stress, attempting to find a suitable balance between stress and growth as follows: triggering accumulation of antioxidant molecules while preventing culture productivity from decay.

## 2. Materials and Methods

### 2.1. Biological Material and Culture Conditions

**Figure 2:**
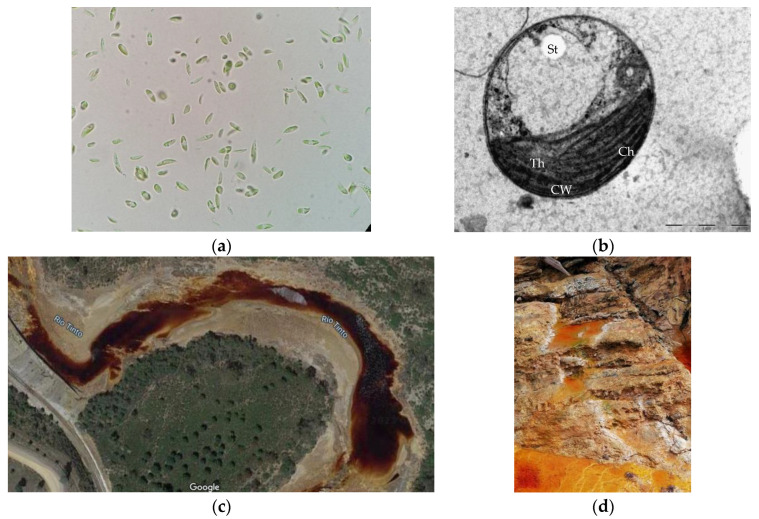
*C. onubensis*: light, 100× amplification (**a**) and transmission electron microscopy (**b**), scale bar 1 μm (×30,000). Abbreviations: Ch, chloroplasts; Th, thylakoids; St, starch grains; CW, cell wall. Tinto River: satellite image of google maps (**c**) and sampling location (**d**).

The organism used for this work was the microalga *Coccomyxa onubensis* ACCV1 (SAG 2510), an acid-tolerant and halotolerant microalga isolated from the acidic waters of the Tinto River (Huelva) in the sampling area at latitude 37.5851153° and longitude -6.550754° (Figure 2a), by the Algae Biotechnology research group (BITAL), of the Department of Chemistry “Professor Jose Carlos Vílchez Martín,” from the Faculty of Experimental Sciences of the University of Huelva [20].

Morphological studies carried out using electron microscopy techniques (Figure 2b) allowed us to determine that *C. onubensis* is a unicellular microalga with an ellipsoidal shape that has a cell wall and a size of approximately 3 μm in length and 2 μm in width. It has a large chloroplast that partially surrounds the nucleus and occupies more than half the cell volume. The nucleus has an approximate size of 1 μm in length and 1 μm in width, while the nucleolus has a diameter of approximately 0.15–0.25 μm, both are located in the central zone of the cell.

*C. onubensis* was grown in an optimized liquid culture medium, K9 [21]. The constituents of K9 (per liter) were as follows: 3.95 g K_2_SO_4_, 0.1 g KCl, 0.5 g K_2_HPO_4_, 0.41 g MgCl_2_, 2.29 g KNO_3_, 0.01 g CaCl_2_, and 5 mL of Hutner traces, which were prepared as described in [5], containing a Fe concentration of 17.98 µM. Fe was used in this study because it is more bioavailable at low pH, mainly because both ionic forms of iron, Fe (II) and Fe (III), are far more soluble (especially ferric iron) at low pH than at neutral or basic pH [22]. In this way, *C. onubensis* cultures were prepared at an initial concentration of, approximately, 0.2 g·L^−1^, from a mother culture in the middle of the linear growth phase, the pH was adjusted to 2.5 and the cultures were subjected to different concentrations of Fe (III), added in the form of FeCl_3_·6H_2_O (VWR, Belgium) from 0 mM (control culture) to 2 mM. The cultures were established in a culture room at 25 °C ± 2. Light was supplied by white light fluorescent tubes, reaching the cultures at a constant light intensity of 150 µmol (photon)·m^−2^·s^−1^ for 24 h, and the cultures were bubbled with air enriched with 2.5% (*v*/*v*) CO_2_.

### 2.2. Light and Transmission Electron Microscopy

Photomicrographs of *C. onubensis* were taken using an Olympus BX-61 microscope (Olympus, Tokyo, Japan) with a CCD Colour-View-II camera (Soft Imaging System, Münster, Germany) and the CellSens analysis imaging system (Olympus, Tokyo, Japan). For transmission electron microscopical observations, the algal cells were collected by centrifugation (5000 rpm at 1957× *g*, 1 min). The algal cells were fixed with 1% glutaraldehyde in 0.1 M sodium cacodylate buffer (pH 7.4) for 2 h at 4 °C. The cells were then washed three times for 5 min using the same buffer. The samples were postfixed with 1% osmium tetroxide in 0.2 M cacodylate buffer at 4 °C for 1 h. Samples were washed with the same buffer, dehydrated in a graded ethanol series and embedded in Epon 812 (Electron Microscopy Science, Hatfield, PA, USA). Ultrathin sections of 80–90 nm obtained by an ultramicrotome (Leica, Wetzlar, Germany) and placed on copper grids were stained with aqueous 1% (*w*/*v*) uranyl acetate and lead citrate. Transmission electron micrographs were observed with a JEM 1011 (JEOL Ltd., Tokyo, Japan) electron microscope using an accelerating voltage of 80 kV. All chemicals used for histological preparation were purchased from Electron Microscopy Sciences.

### 2.3. Growth and Photosynthetic Viability

Productivity, the increase in biomass in a culture over time, was calculated through the following equation:

$$Productivity\;\left(g\cdot L^{-1}\cdot d^{-1}\right)=\frac{C_{t}-C_{0}}{t_{t}-t_{0}}$$

where $C_{t}$ and $C_{0}$ represent the cell density for times *t* and zero. Cell density was determined by measuring the dry weight (dw) of the biomass contained in 2 mL of culture medium.

Photosynthetic viability was determined based on the chlorophyll fluorescence measurements, the maximum quantum yield (Fv/Fm) of Photosystem II (PSII) according to published methods [23]. Photobiochemical parameters were determined through the OJIP protocol. Culture samples were diluted so that all Chl fluorescence measurements (Chl, chlorophyll) were performed on samples with the same cell concentration, OD_750_ = 0.2 (OD_750_, optical density at 750 nm). Quantum yield (Qy) was measured by placing *C. onubensis* culture samples into the measuring chamber of portable pulse amplitude-modulated fluorimeter model AquaPEN AP-C 100 (Photon Systems Instruments, Drásov, Czech Republic). Fv represents the minimum level of fluorescence observed after exposing the cells to a non-actinic beam and acclimatizing them in the dark for 10 min, while Fm represents the maximum fluorescence observed in cells after exposing them to a short but saturating actinic light pulse.

### 2.4. Chlorophyll and Carotenoid Determination

Chlorophyll and carotenoid content were determined as described by [24]. Culture samples, containing 1 or 2 mL, were centrifuged at 4400 rpm for 5 min, methanol was added to the pellet and the mixture was placed in an ultrasound bath (60 °C, 5 min) to weaken the microalgal cell wall. After another centrifugation step, the supernatant was collected and analyzed by UV/Visible spectrophotometry. Modified Arnon’s equations were used to calculate the chlorophyll and carotenoid concentrations in the extracts. The extracts obtained were used to analyze the antioxidant capacity of the microalga and the determination of polyphenols compounds.

For specific carotenoid analysis and quantification, separation was performed by liquid chromatography (HPLC; Beckman System gold) using an RP-18 column with a flow rate of 1 mL/min and injection extract volume of 40 µL. The applied gradient was the following (solvent A; ethyl acetate and solvent B; acetonitrile/water, 9:1 *v*/*v*): 0–16 min, 0–60% solvent A; 16–30 min, 60% A; 30–35 min, 100% A. In order to quantify, pigment standards supplied by DHI-Water and Environment (Denmark) were injected. Quantification of the selected pigments was based on comparison of peak areas obtained from methanolic extracts of *C. onubensis* with those areas obtained from the injected standards.

### 2.5. Antioxidant Capacity

Antioxidant capacity of the methanolic extracts of microalga was determined by the modified version of the DPPH (2,2-diphenyl-1-picryl-hydrazyl-hydrate) free radical method described by [25]. The antioxidant capacity was determined by the decrease in absorbance at 515 nm of a methanolic solution of DPPH in the presence of the different methanolic samples of the microalga. A concentrated solution of DPPH (Sigma Aldrich, Darmstadt, Germany) in methanol of approximately 0.4 g·L^−1^ was prepared and diluted with methanol to obtain an absorbance around 0.8. Next, 950 µL of the diluted DPPH solution was made to react with 50 µL of the methanolic sample. The absorbance at 515 nm at time 0 was then measured in a UV-vis spectrophotometer model Evolution 201 (Thermo Fisher Scientific, Walthman, MA, USA) in a glass cuvette, using methanol as a blank. Subsequently, the samples were allowed to stand for 30 min at room temperature and the absorbance of the sample at 515 nm was measured after that time. Trolox (Fisher Scientific, Walthman, MA, USA) was used as an external standard. The antioxidant capacity was determined through the difference in absorbance at time 0 and time 30 min and was expressed as μmol eq-Trolox·g^−1^ biomass.

### 2.6. Polyphenols Determination

Total polyphenols were determined using the procedure described by [26]. According to this, phenolic compounds were oxidized by the Folin–Ciocalteu reagent (Panreac, Barcelona, Spain), resulting in a blue color. The reaction was carried out in an alkaline medium; for this, sodium carbonate was added to the samples and the absorbance was measured at 725 nm by UV-vis spectrophotometry model Evolution 201 (Thermo Fisher Scientific, Walthman, MA, USA). Total content of polyphenols was obtained using a calibration line from a standard solution of gallic acid 1-hydrate (Panreac, Barcelona, Spain), and was expressed as mg-eq· gallic acid·L^−1^.

Flavonoid compounds were determined by a spectrophotometric method described by [27]. For this, the following reagents were used: NaNO_2_, AlCl_3_·6H_2_O and NaOH. As an external standard, a 1 g·L^−1^ catechin stock, (+)-catechin hydrate (Sigma Aldrich, Darmstadt, Germany) in MilliQ water was used. The absorbance value was measured at 510 nm using UV-vis spectrophotometry model Evolution 201 (Thermo Fisher Scientific, Walthman, MA, USA). Total content of flavonoids was calculated by extrapolating the values obtained in the calibration line.

### 2.7. Superoxide Dismutase

Superoxide dismutase (SOD) activity was tested as described by [28], monitoring the inhibition of nitroblue-tetrazolium (NBT) staining at 530 nm due to the decrease in superoxide associated with SOD activity. NBT reduced superoxide anion and resulted in a pink color that can be measured at 530 nm. Each unit of SOD was defined as the amount of enzyme required to inhibit 50% of the reaction of superoxide anion with NBT.

The first stage consisted in the preparation of the algal crude extract, for which algal cultures were harvested during the exponential growth phase by centrifugation at 4400 rpm model Eppendorf centrifuge 5702 (Thermo Fisher Scientific, Walthman, MA, USA). The pellet was resuspended in 2 mL of extraction buffer per g of fresh weighted pellet. The SOD-extraction buffer contained 50 mM P-Buffer + 1 mM PMSF + 0.1 mM EDTA + 1% PVP. Pellet was washed 3 times with the extraction buffer, and cells were disrupted, for which the ball mill method model mm 400 (Retsch, Haan, Germany) was used applying 10 cycles of 30 s with a 1-min rest between them. Next, the mixture was spined for 10 min in a refrigerated centrifuge at 4 °C at 16,000× *g* model FC5816R (Ohaus, Nänikon, Switzerland).

The enzyme activity was determined spectrophotometrically, following the consumed H_2_O_2_ decay at 530 nm (ε = 2.8 mM^−1^ cm^−1^) and 25 °C. The reaction mixture contained, per milliliter: 0.1 Tris-HCl 500 mM, 0.5 EDTA 10 mM, 0.05 Xanthene 10 mM, 0.05 NBT 1 mM, 0.04 μL Xanthene Oxidase, 0.194 H_2_O. A blank sample was prepared accordingly, but NBT was replaced by demi water. One unit, U, of activity represents the enzyme required to consume H_2_O_2_ at 1 μmol·min^−1^. Specific activity of SOD was calculated considering the difference absorbance between the sample and blank expressed as a U·mg^−1^ of proteins.

### 2.8. Proteins

Proteins content was determined spectrophotometrically by the Lowry quantification method, by interpolation on a bovine serum albumin (BSA) standard curve, measured at 580 nm [29]. Reagent A (2 g Na_2_CO_3_ in 100 mL NaOH 0.1 M), reagent B (0.1 g CuSO_4_·5H_2_O in 10 mL H_2_O) and reagent C (0.2 g KNaC_4_H_4_O_6_·4H_2_O in 10 mL H_2_O) were prepared. Crude extracts prepared for SOD activity were diluted with SOD-extraction buffer. More details of SOD reagent can be found in Section 2.7 of Material and Methods. Next, 5 mL of reagent D (reagent A:B:C 100:1:1 *v*/*v*) was added to each sample, and a dark incubation for 15 min was performed. It was followed by the addition of 0.5 mL of reagent E (Folin–Ciocalteu reagent diluted with H_2_O (1:3) and dark incubation for 30 min. The calibration curve was prepared from aqueous solutions obtained from 0 to 277 µg·L^−1^ of BSA (Sigma Aldrich, St. Louis, MI, USA). Finally, the protein content of the samples was determined spectrophotometrically with absorbance measurement at 580 nm, using distilled water as a blank. Quantification of proteins was based on interpolation on the standard curve (y = 0.031x + 0.0504, R^2^ = 0.9952).

### 2.9. Lipids and Fatty Acids

Lipid composition was measured in lyophilized biomass samples as described by [30]. Briefly, 10 mg of sample was extracted with chloroform:methanol (2:1, *v*/*v*). The extraction process was followed by solvent evaporation using an N_2_ stream. Subsequently, lipid content was determined gravimetrically.

The fatty acid analysis was performed according to the following procedure. Once the acylglycerides were extracted, the corresponding fatty acids methyl-ester (FAME) were obtained through acid catalysis-mediated transesterification. The mixture was heated at 85 °C for 1 h, then cooled and washed with hexane and water. The FAMEs were separated by centrifugation (2500 rpm, 10 min). FAMEs were separated and analyzed in a gas chromatograph system equipped with flame ionization detector (model 7890A, Agilent, California, USA). A 1-μL sample was injected into a silica capillary column (30 m, 0.32 mm id and 0.25-μm film thickness). Helium was used as carrier gas. The applied flow rate was 1.5 mL·min^−1^ and the split ratio 20:1. The injector and detector temperature values were 100 °C and 220 °C, respectively. The programmed oven temperature raised from 80 to 140 °C at 5 °C min^−1^, followed by increase to 170 °C at 4 °C min^−1^ and then maintained for 2 min at 170 °C. Temperature was then increased to 190 °C at 1 °C min^−1^ and maintained for 2 min. The final temperature in the oven was 210 °C. Each one of the FAME peaks was identified by comparing retention times with those of mixed fatty acids standards (FAMEs Mix C4-C24 Supelco Analytical). Concentrations of FAMEs (ppm) were quantified by comparing their peak areas with those obtained from the standards of known concentration (Sigma Aldrich, St. Louis, MI, USA). Fatty acid composition was calculated as percentage of the total fatty acids in the volume of hexane. For quantification, tripentadecanoin was used as internal standard (Sigma Aldrich, St. Louis, MI, USA).

### 2.10. Statistics

Unless otherwise indicated, the presented data are the means of three independent experiments and the standard deviations are represented in the corresponding figures and tables. The data from the different treatments were analyzed with univariate statistical models using analysis of variances (ANOVA) with a confidence of 95%. The analysis was performed using Minitab 17 software.

## 3. Results

Fe (III), as already described, plays a determining role in physiological processes and is an inducer of the antioxidant response through its direct participation in the Fenton reaction. The effect that the addition of Fe (III) can exert on the productivity and photosynthetic viability of *C. onubensis,* along with the accumulation of antioxidant molecules, was analyzed. The molecules selected for the study are of nutraceutical value and were described to display anti-inflammatory activity.

### 3.1. Abiotic Stress on Cell Growth and Photosynthetic Viability

Initially, the effect of Fe (III) on the growth and photosynthetic viability of cultures of the acidophilic microalga was analyzed in order to determine the range of Fe (III)-sublethal concentrations. To study how excess Fe (III) affects the microalga *C. onubensis*, cultures were supplemented in Fe (III) from 0 to 2 mM.

**Figure 3:**
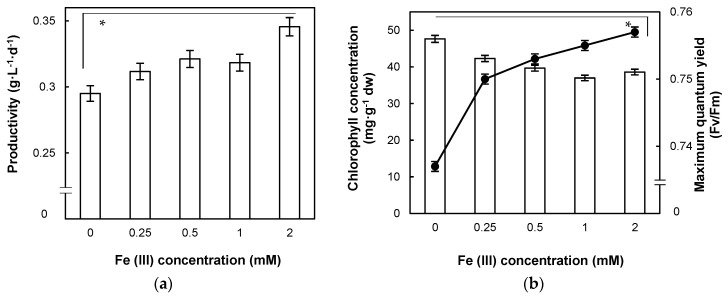
(**a**) Microalgal productivity, (**b**) intracellular chlorophyll concentration (bars) and maximal photosynthetic efficiency of photosystem II (Quantum yield, solid circles), data corresponding to day 4 of cultivation, as a function of the Fe (III) concentration in cultures of the microalga *C. onubensis*. (*) Represents the significant differences of all treatments with respect to the control cultures with 95% confidence.

The difference in growth between cultures was determined based on their productivity (Figure 3a). The physiological status of the microalgal cultures is connected with the photosynthetic cellular viability that can be determined as the photosynthetic efficiency of photosystem II (PSII), measured as Quantum yield (Qy), and the total chlorophyll (Chl) concentration of the cultures (Figure 3b).

Figure 3a shows the quantitative difference in the biomass productivity of *C. onubensis* cultures as a function of the Fe (III) concentration added. The increase in Fe (III) resulted in increased productivity of the cultures, reaching a maximum productivity of 0.35 g·L^−1^·d^−1^, 17% higher than that of the control culture, in the 2 mM Fe (III)-added culture. The results shown in Figure 3a corresponds to day 4 of the experiment and intend to reflect the different effect of Fe (III) in the cultures depending on the Fe (III) concentrations used during the linear growth phase. The effect of Fe (III) on each culture growth throughout the experiment is shown in Figure S1 of Supplementary Material. The cultures with increased Fe (III) levels showed increased biomass concentration values.

Figure 3b shows the effect of Fe (III) on the photosynthetic viability of *C. onubensis* cultures. As shown, increasing Fe (III) concentrations resulted in decreased intracellular concentration of chlorophyll, which became 19% lower for the 2 mM Fe (III) culture compared to the control culture, while a higher efficiency in the photochemical process at PSII is achieved, being 27% higher in the 2 mM Fe (III) culture than that of the control culture. The time-course variation of the intracellular chlorophyll concentration as a function of the Fe (III) concentration is shown in Figure S2 of Supplementary Material. A decrease in the cell chlorophyll content is observed with increased Fe concentrations in cultures of *C. onubensis*. The time-course variation of Chl a and Chl b along the experiment time in cultures exposed to Fe (III) is shown in Figure S3 in the Supplementary material. Figure S3b shows decreasing Chl b contents along the experiment time (from day 0 to day 9) in cells exposed to increased Fe (III) levels, while the trend for Chl a content does not correlate to Fe (III) concentration as clearly as Chl b.

For a better understanding of the Fe (III) role during the photochemical process, samples of Fe (III)-added cultures were subjected to Chl fluorescence analysis. Table 1 illustrates some selected parameters, which were calculated based on the OJIP Chl fluorescence signals.

Table 1 shows selected photochemical parameters of *C. onubensis* that express specific electron and energy fluxes per reaction center (RC), as described in the Table legend. Among the parameters listed, the net closing rate of RCs during illumination (Mo) decreased as Fe (III) increased, being 22% lower for the higher Fe (III) concentration tested. The electron transport flux per RC (ETo/RC) increased by 5.4% for the 2 mM Fe (III) culture with respect to the control culture, while the trapping energy flux per RC (TRo/RC) remained almost stable. ABS/RC expresses the absorption flux per RC, related to the apparent antenna size, which decreased slightly when Fe (III) is added to the cultures, becoming 4% lower for the 2 mM Fe (III) culture with respect to the control culture. The dissipated energy flux per RC (DIo/RC) decreased as Fe (III) increased, being 11% lower for the 2 mM Fe (III) culture with respect to the control culture.

The results obtained seem to evidence the expected Fe (III) involvement in the photosynthetic redox processes. Conversely, Fe (III) is directly involved in the so-called Fenton reaction, which triggers the production of reactive oxygen species within the cell. Therefore, the antioxidant response of the microalga to cope with the oxidative stress generated through the addition of Fe (III) to the cultures was analyzed.

### 3.2. Antioxidant Cell Response as a Function of the Fe (III) Concentration

**Figure 4:**
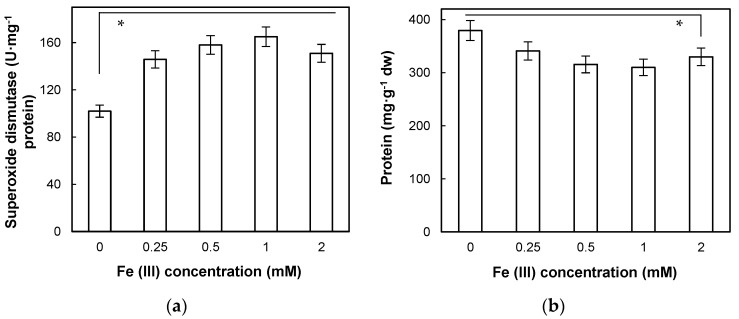
Effect of Fe (III) on the SOD enzyme activity (**a**) and total cell protein content (**b**) of *C. onubensis* cultures. The data presented correspond to day 11 of cultures incubation in Fe (III), last day of experiment. (*) Represents the significant differences of all treatments with respect to the control with 95% confidence.

As described in the Introduction section, superoxide dismutase (SOD) is one of the key enzymes in the defense mechanisms against oxidative stress. Figure 4 shows the evolution of SOD activity as a function of the Fe (III) concentration supplied to *C. onubensis* cultures.

Figure 4a shows the evolution of the specific SOD activity as a function of the Fe concentration added to cultures of the microalga *C. onubensis*. As shown, the incubation under increasing Fe (III) concentrations resulted in increased specific SOD activity levels, this being maximal for the culture supplied with 1 mM Fe (III), approximately 62% higher than that of the control culture.

Figure 4b shows the changes in the total cellular protein concentration as a function of the Fe (III) concentration added to *C. onubensis* cultures. According to the data, as Fe concentration supplied to the cultures increased a slight decrease in cell protein concentration was found, this being minimal for the 1 mM culture, 18% lower than that value obtained from the control culture. The results suggest an increased expression of the antioxidant biochemical response under increased Fe-mediated oxidative pressure, in parallel to an apparently less active protein biosynthesis, which seems not to compromise the photosynthetic viability, as shown in Figure 3.

**Figure 5:**
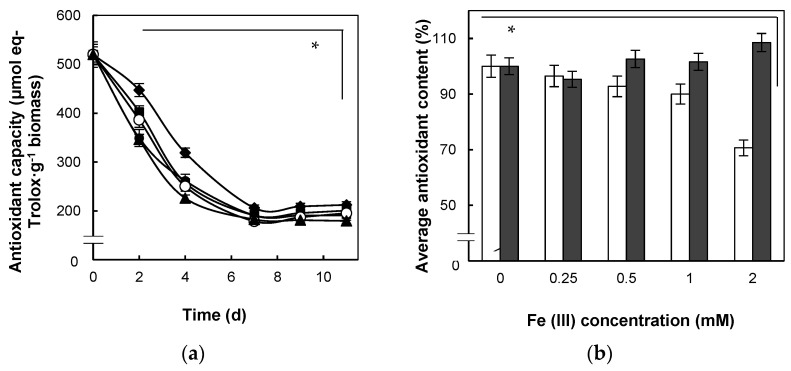

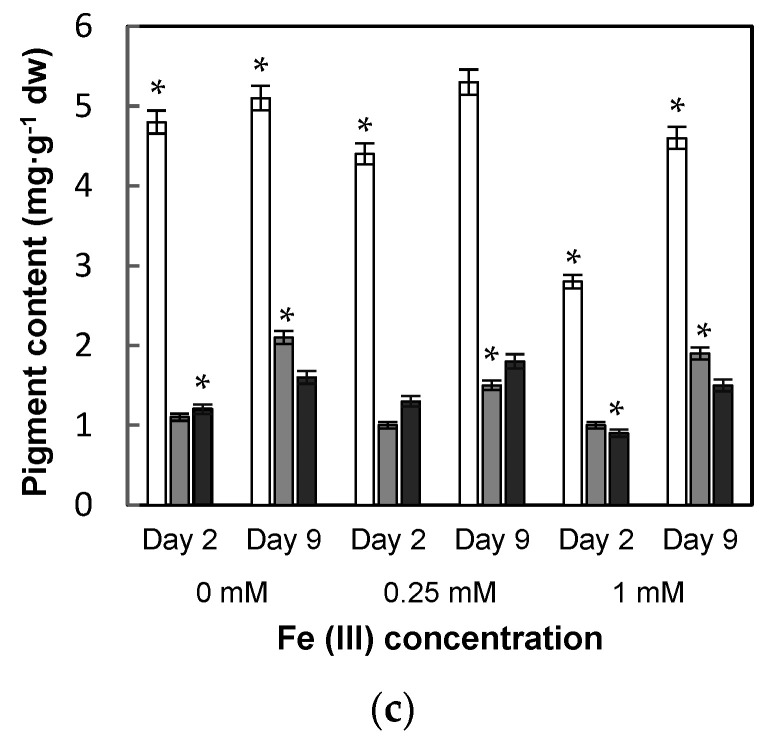
Effect of Fe (III) concentration on the non-enzymatic antioxidant response of *C. onubensis* cultures. (**a**) Symbols for Fe (III) concentration: 0 (-◆-), 0.25 (-◼-), 0.5 (-●-), 1 (-○-) and 2 (-▲-) mM; (**b**) Average antioxidant content between days 2 and 9 of cultivation: polyphenols (empty bar) and flavonoids (black bar). In total, 100% of polyphenols and flavonoids corresponds to an average content of 17.50 and 4.75 mg·g^−1^ biomass, respectively; (**c**) Pigment content: lutein (empty bar), neoxanthin (grey bar) and beta-carotene (black bar) for day 2 and 9 of cultivation. (*) Represents the significant differences of all treatments with respect to the control with 95% confidence.

In this study, the total antioxidant capacity of extracts from the Fe (III)-added cultures was used as an indicator of the impact of Fe (III) on the cell antioxidant molecular pool. The results are shown in Figure 5a. In addition, the eventual accumulation of antioxidants was assessed by measuring the average content throughout the experiment of major antioxidant compounds, including polyphenols, carotenoids and flavonoids (Figure 5b), as a function of the Fe (III) concentration.

Figure 5a represents the antioxidant capacity of culture samples of the acidotolerant microalga cultures subjected to different Fe (III) concentrations. As shown, Fe induced changes in the antioxidant capacity of the microalga. The increase in Fe concentration caused a decrease in the antioxidant capacity, which was 22% lower for the culture with the maximum Fe (III) concentration tested—2 mM—on day 2 of the experiment, with respect to the control culture.

The Fe-dependent decrease in antioxidant capacity should accordingly indicate a reduction in the antioxidant molecules pool of *C. onubensis* upon a long-term exposition to the metal. As shown in Figure 5b, polyphenol content in the cells apparently decreased as the Fe concentration increased, with minimum values for the 2 mM Fe (III) culture, approximately 30% lower than those for the control culture. On the other hand, the intracellular flavonoid content tends to increase as the Fe concentration increased, reaching a maximum value for the maximum Fe (III) concentration tested—2 mM—, this being 8% higher with respect to the control culture. The cell content of polyphenol compounds and flavonoids remained roughly unaltered during the first 24 h of the experiment, then started to decrease (polyphenols) an increase (flavonoids) slightly, respectively, from day 2. For this reason, Figure 5b shows the averaged content of both molecules in the above-referred time period, from day 2 to day 9.

Figure 5c shows the variation in the content of the majority of carotenoids of *C. onubensis* produced during days 2 and 9 of cultivation as a function of the Fe (III) concentration. Short-term cultivation, 48 h, resulted in a slight decrease in the lutein content compared to control cultures, as Fe (III) concentration increased, while under long-term cultivation, a recovery of that content was obtained. Slight differences were found in neoxanthin and beta-carotene in Fe-added cultures. Nevertheless, under long term cultivation an increase of about 30–50% was obtained for neoxanthin and beta-carotene. Day 2 of the experiment was taken as a reference, based on the fact that the carotenoid content in control cultures did not change in the first 24–48 h days, and the relevant variation in Fe (III)-supplement cultures occurred from day 2.

The results obtained show that Fe (III) has a direct effect on the enzymatic and non-enzymatic antioxidant responses of the microalga. The main trend inferred from the results points to an imbalanced variation of the majority of antioxidant molecules.

### 3.3. Triggering the Accumulation of Fatty Acids and Lipids

In this section, the effect of Fe (III) on the accumulation of lipids and fatty acids in the microalga *C. onubensis* as a response to the imposed oxidative stress was analyzed.

**Figure 6:**
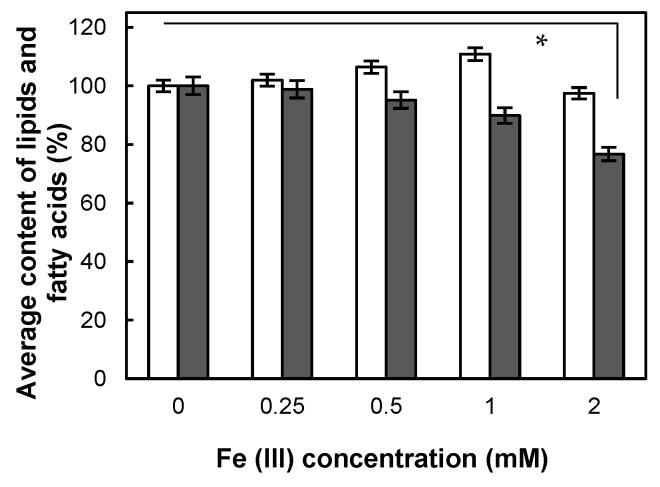
Effect of Fe (III) concentration on the average content between days 2 and 9 of lipids (empty bars) and fatty acids (grey bars) of the microalga *C. onubensis*. In total, 100% of average content of lipids and fatty acids corresponds to 31.52 and 12.63 mg·g^−1^ biomass, expressed as percentage, respectively. (*) Represents the significant differences of all treatments with respect to the control with 95% confidence.

Figure 6 shows the variation in the whole cell content of lipids and fatty acids in *C. onubensis* biomass samples as a function of the Fe (III) concentration of the cultures. According to the data, the whole cell lipid content for cultures grown in the presence of Fe shows a slight increase, being maximal for the 1 mM Fe (III) culture, approximately 11% higher than that of the control culture. On the other hand, the whole cell content of fatty acids tended to decrease when the Fe (III) concentration increased, the lowest fatty acids content (23% lower than that contained in control culture samples) being reached in the culture with the maximum Fe (III) concentration—2 mM.

**Figure 7:**
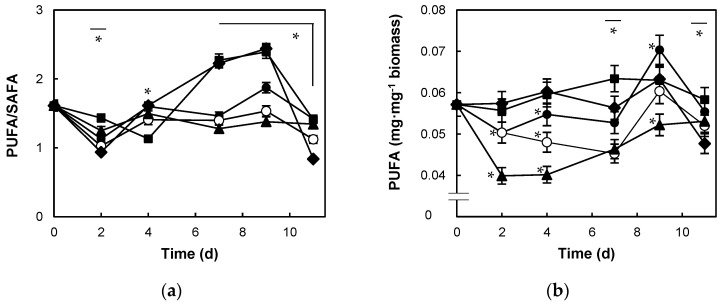
Effect of Fe (III) concentration on the time-course evolution of fatty acids saturation level. (**a**) PUFA/SAFA ratio and (**b**) PUFA content of the microalga *C. onubensis.* SAFA: saturated fatty acids; PUFA: polyunsaturated fatty acids. Symbols for Fe (III) concentration: 0 (-◆-), 0.25 (-◼-), 0.5 (-●-), 1 (-○-), and 2 (-▲-) mM Fe (III). (*) Represents the significant differences of all treatments with respect to the control with 95% confidence.

As discussed further in this study, the ROS-neutralizing role of unsaturated fatty acids might partly explain their content variation in cultures exposed to Fe (III). Consequently, the fatty acids profile of *C. onubensis* samples from cultures exposed to Fe (III) was analyzed. The results are shown in Figure 7.

Figure 7 shows the time-course evolution of the whole cell content of unsaturated fatty acids in *C. onubensis* cultures as a function of the Fe concentration. According to Figure 7a, the ratio of polyunsaturated fatty acids to saturated fatty acids tended to remain roughly stable for several days after the experiment started. From day 4, an increase in the PUFA/SAFA ratio was obtained in the control culture and in the culture added with 0.25 mM of Fe (III), while the values in the other cultures remained almost stable.

Specifically, the abundance of polyunsaturated fatty acids was plotted in Figure 7b. The whole cell PUFA content tended to decrease during the first days of incubation as the concentration of Fe supplied to the cultures increases, except for the 0.25 mM culture that presented similar values to those of the control culture. From the point of view of the physiological significance of the results, those data obtained during the early growth phase, until day 3–4 of cultivation, should be more relevant to the final conclusions of the study, as all the microalgal cells were experiencing most of the total Fe (III) concentration added to the cultures, unlike at later stages of growth, above day 7 of cultivation.

The maximum whole-cell fatty acids content accumulated by *C. onubensis* as a function of the Fe concentration was analyzed, and the main data are shown in Table 2.

Table 2 shows the maximum whole cell content of fatty acids, expressed as a percentage with respect to the dry weight of the biomass, obtained from *C. onubensis* culture samples. The data correspond to the accumulation of main fatty acids as a function of the Fe concentration. The data exceeding those obtained from control culture samples are highlighted in bold. According to the Table, the presence of Fe in *C. onubensis* cultures resulted, on the one hand, in a decreased content of saturated fatty acids C16:0 and C18:0. On the other hand, in the case of unsaturated fatty acids, an increase was observed for low concentrations of Fe (III), while their content tended to decrease as Fe concentration increased. It should be noted that the C18:2 and C18:3 fatty acids, which correspond to omega 6 and omega 3 series, respectively, reached maximum values in Fe (III) added cultures: at 0.5 mM, C18:2 content became 48% higher than that value obtained in the control culture, and at 0.25 mM C18:3 concentration was 36% higher than that of the control culture.

Saturated fatty acids are adequate to obtain biodiesel, while fatty acids from the omega-3 and omega-6 families are considered essential in nutrition and have a high value in human health. In this way, the decrease in saturated fatty acids C16:0 and C18:0 under low Fe levels, together with the increase in polyunsaturated fatty acids from the omega 6 and omega 3 families, C18:2 and C18:3, respectively, would result in fatty acids, rich microalgal biomass that could be suitable as a supplement for animal nutrition and/or the design of functional foods.

## 4. Discussion

### 4.1. Abiotic Stress on Cell Growth and Photosynthetic Viability

Fe plays a key role in the cellular biochemistry of microalgae due to its redox properties, and, linked to the latter, Fe is involved in fundamental processes such as photosynthesis, respiration, nitrogen fixation and DNA synthesis [31,32]. Our results in *C. onubensis* are in a good agreement with this fact showing that Fe (III), added to the cultures in a certain concentration range, stimulates the growth of the acidotolerant microalga (Figure 3). The range of Fe (III) concentrations used in this study still is below toxic levels since the highest concentration of Fe (III) added to the cultures resulted in the highest growth of the microorganism.

Photosynthesis is the most Fe-requiring process in plants. The photosynthetic electron transfer chain converts light energy into chemical energy between photosystems II and I (PSII and PSI) via cytochrome (cyt) b6f in thylakoid membranes [33]. During this process, most of the Fe taken by microalgae from the medium distributes into [4Fe-4S] cluster-containing PSI proteins and Fe-containing cyt b6f complexes. Consequently, Fe mediates light-driven electron transfer in microalgal cells [34,35]. Fe–S cluster-dependent metabolism in microalgae is sensitive to the decreased bioavailability of ferric iron at neutral and basic pH, as well as to the direct oxidation of sulfur intermediates and Fe–S clusters by reactive oxygen species [36]. Fe is more bioavailable at low pH, mainly because both ionic forms of iron, Fe (II) and Fe (III), are far more soluble (especially ferric iron) at low pH than at neutral or basic pH [22]. In addition, in the acidic natural habitat of *C. onubensis*, Fe (III) is by far the predominant form of iron due to the rapid, natural oxidation of Fe (II) to Fe (III).

In this study, the microalga *C. onubensis* was cultivated under acidic conditions with a supplement of Fe (III). The higher Fe (III)-bioavailability under acidic conditions should result in increased Fe (III)-intracellular levels, including Fe (III) presence in inorganic Fe-S clusters. In this sense, the results obtained for the photobiochemical parameters (Table 1) show that the addition of Fe (III) to the cultures favors the electron transport flux (ETo) while the net closing rate of RC (Mo) decreases. As Mo = Tro–ETo, and the trapping energy flux per RC (TRo) remains almost stable, the reduction in the net closing rate of RC means that the electron transport chain (ETC) should be more active in Fe-added cultures. The increase in the efficiency of PSII (Figure 3b), together with the obtained enhancement in the ETC (Table 1), allows us to suggest that photosynthesis in *C. onubensis* becomes stimulated by moderated concentrations of Fe (0.25–2 mM).

Fe-mediated increased photosynthetic capacity addresses faster growth of *C. onubensis*. In batch cultures, this fact led to a faster increase in the cell turbidity that could favor the cells to experience light limitation. Thus, the subsequent reduction in the chlorophyll cell content (Figure 3b) could be understood as a light-capturing apparatus adaptation strategy to reduce the shadowing effect among the cells [37]. In this sense, the addition of Fe (III) to *C. onubensis* seems to result in an apparent reduction of the antenna size. This fact is in good agreement with the values obtained for the photobiochemical parameters determined in Fe-exposed cultures, shown in Table 1; the decrease in ABS/RC and DIo means less energy absorption per reaction center rate (ABS/RC) and less non-photochemically dissipated energy per reaction center (Dio), which results in minimized losses in light use efficiency in a light-limited culture. In this scenario, the decreased chlorophyll cell content could be interpreted as an apparent reduction of the antenna size per cell, expressed to increase light capture in dense cultures. In this sense, the decreased Chl b contents along the experiment time (from day 0 to day 9) shown in Figure S3b for cells exposed to increased Fe (III) levels is in line with the discussion. As published, a reduction of chlorophyll b can result in a reduction of light-harvesting complex proteins stability, which becomes degraded. This fact results in the reduction of the apparent optical cross-section of the light-harvesting antenna [38]. The same reduction effect in the antenna size is hypothesized based on the results obtained in our experiments for *C. onubensis* cultures exposed to Fe (III). This strategy has been described by other authors in cultures under oversaturated light irradiance as a means to decrease the non-photochemical quenching aimed at reducing photooxidative damage in the chloroplasts [37]. For practical purposes at a large scale, the addition of Fe to *C. onubensis* cultivation systems might be considered to modulate cell growth and chlorophyll cell content.

### 4.2. Antioxidant Response as a Function of the Fe (III) Concentration

The results obtained showed that the presence of Fe (III) in *C. onubensis* cultures triggers biochemical responses, which are typically expressed as a defense mechanism against reactive oxygen species, particularly, the increased activity of the enzyme superoxide dismutase (Figure 4a) evidenced Fe (III)-mediated increased oxidative stress. At higher concentrations of Fe (III), the expression of genes encoding the activity of antioxidant enzymes is induced, including ascorbate peroxidase, superoxide dismutase, dehydroascorbate reductase, and catalase [17,18]. Nevertheless, the enzyme superoxide dismutase has a constitutive presence according to scientific information [39] and is involved in the cellular antioxidant response, being responsible for catalyzing the transformation of O_2_^•^¯ into H_2_O_2_. According to the following equation:

2 O_2_^•−^ + 2H^+^ → 2 H_2_O_2_ + O_2_

Reactive oxygen species have been described to cause protein oxidation during oxidative stress [15]. In this way, the decrease in the protein content observed as the Fe concentration increases (Figure 4b) could theoretically be assigned to the damage caused by the greater activity of the reactive oxygen species generated by the oxidative stress caused by the addition of Fe to the cultures. However, it should again be emphasized that this decrease in protein content could be compatible with the increase in growth observed in the presence of Fe (III). As described later, this means that increasing the number of cells limits light absorption in the culture by increasing the shadowing effect, thus reducing turbidity to make light more available through a reduction in the chlorophyll cell content. This should be accompanied by a reduction in the protein content and, thus, in the light-harvesting complex proteins in coherence with the obtained results.

The slightly increased photosynthetic electron flux (ETo, Table 1) should result in a slightly increased reduced ferredoxin pool required as a physiological electron donor to a number of assimilatory enzymes, including GOGAT (glutamate synthase) for nitrogen assimilation leading to amino acid biosynthesis [40]. Accordingly, photosynthetically produced NAD(P)H availability for assimilatory metabolism should be greater. This is in good agreement with the enhanced growth data obtained in Fe (III)-added cultures. In this context, the slightly lower protein content of Fe (III)-added cultures might be, partly, a consequence of a more intensive carbohydrate and/or lipid biosynthesis in Fe (III)-added cultures (the latter shown in Figure 5 and Figure 6), thus shifting the protein to carbohydrate and lipid ratio slightly in favor to the latter energy storage molecules, among others [41].

It has been described that microalgae respond to high concentrations of metals by inducing the activity of different antioxidant systems, including increased activity of enzymes such as superoxide dismutase, catalase, glutathione peroxidase and ascorbate peroxidase, and the synthesis of low molecular weight compounds, glutathione and carotenoids among them [17]. Some of the results obtained in this study apparently evidenced lower antioxidant capacity for cell extracts from Fe (III)-supplemented cultures (Figure 5a). There is a certain decrease in said capacity as a direct function of the added Fe (III) concentration, particularly, during the first days of culture, when most of the Fe (III) could presumably be bioavailable. The apparently lower antioxidant capacity should correspond to cultures under weaker oxidative stress. Nevertheless, the increase in the SOD activity (Figure 4a) as the Fe (III) concentration increases might be a sign of a more active antioxidant response of the microalga, though a more extensive analysis of other antioxidant enzymes (ascorbate peroxidase, glutathione reductase, catalase, etc.) is required to define the antioxidant response more accurately. This scenario is coherent with a greater involvement of polyphenols in ROS-neutralizing reactions (Figure 5b), which would explain their apparent decreased content in presence of Fe (III). Thus, conversely to what many times is accepted, an apparent lower antioxidant capacity might be compatible with the decrease in the chemically active antioxidant form of antioxidant molecules, which can eventually be not detected through their specific standard determination methods. For instance, maximal absorption wavelengths for carotenoid and phenol compounds can shift due to their double bounds' oxidation through reaction with oxidizing agents, as elegantly described by [42]. This would affect chromatographic retention times and maximal light absorption of target compounds, then proving the complexity of accurately determining any antioxidant molecule content in any extract. Indeed, the decrease in the lutein content when Fe (III) is more bio-available together with the lutein content recovery obtained under long-term cultivation (Figure 5c) suggests the existence of carotenoid-based regulation mechanisms in response to the oxidative stress generated. In this sense, lutein biosynthesis in *C. onubensis* seems to be constitutive as the contents in Fe (III)-added cultures do not largely differ from the content in control cultures, 4–5 mg·g^−1^ dw.

Flavonoids increase their intracellular content in the presence of Fe (III), which in principle, allows us to suggest a direct relationship between the generation of oxidative stress and the expression of the biosynthesis of these molecules in acidophilic microalgae. Flavonoids are considered a secondary system for the elimination of ROS present in the Fenton reaction already mentioned in the Introduction section. These molecules also help eliminate oxidative species formation risk caused by excess energy from photosynthesis [43] through the so-called auxiliary electron transport (AET) pathways [44]. AET pathways are comprised of electron sinks that function as efficient release valves for excessive electrons in the ETC, any of them with the ability to reduce O_2_ to H_2_O—without the concomitant production of ROS known as the Mehler-like reaction [45]—a sort of chloroplast respiration.

Antioxidant molecules are commonly biosynthesized in microalgae to cope with oxidative stress and help maintain the homeostatic balance in the cultures. Several studies showed that the antioxidant capacity of microalgae, including cell content in flavonoids and/or polyphenol compounds, increased in cultures with a high concentration of heavy metals [46,47].

### 4.3. Stimulation of the Accumulation of Fatty Acids and Lipids

As commented in the above section, Fe has strong prooxidative properties [48], and microalgae such as *C. onubensis*, which grows naturally in the presence of high concentrations of this metal, could express antioxidant responses in Fe (III)-rich media. In response to this stress, terpenoid compounds (carotenoids) and unsaturated fatty acids tend to accumulate in microalgae [49]; thus, an increase in lipid concentration would be expected in the acidotolerant microalga cultures treated with Fe. The results obtained are coherent with this reasoning (Figure 6). Nevertheless, our results evidence a slight decrease in the whole cell content of fatty acids in Fe (III)-added cultures. We cannot offer a clear explanation to this fact, though the decrease in fatty acids might be linked to the use of stored energy in stressed cultures [50].

As discussed in the Introduction section, Fe is an essential bioelement but highly toxic in high concentrations due to its ability to exchange electrons with various substrates, giving rise to ROS. The generation of hydroxyl radicals produces oxidative stress as a consequence of their reaction with lipids, proteins and nucleic acids, resulting in irreversible damage to cell structures [15]. Some of these damages include loss of lipid membrane permeability when the hydroxyl radical reacts with the unsaturated fatty acids of membrane phospholipids, and thus the unsaturated character of membrane lipids, which is required to keep membrane fluidity, is lost [16].

The results obtained are coherent with this fact (Figure 7). As the concentration of Fe supplied increases, the whole cell content of unsaturated fatty acids decreases, probably in response to the oxidative stress produced. Thus, ROS are expected to react with unsaturated fatty acids, which partially lose their unsaturation level through lipid peroxidation, giving rise to conjugated dienes, lipids with peroxyl radicals and hydroperoxides [51]. However, the eventual decreased abundance of membrane unsaturated fatty acids in *C. onubensis* does not lead to a loss of cell activity, quite the contrary according to the growth results obtained in the presence of increasing concentrations of Fe (III).

The variation in the fatty acids profile for the microalga *C. onubensis* when subjected to Fe (III) is shown in Table 2. Saturated fatty acids are used to obtain biodiesel, while fatty acids from the omega-3 and omega-6 families are considered essential and have a high value in human health. In this way, the decrease in saturated fatty acids C16:0 and C18:0, together with the slight increase in polyunsaturated fatty acids of the omega 6 and omega 3 families, C18:2 and C18:3, respectively, for cultures treated with low Fe (III) levels would result in biomass that could be suitable for animal nutrition and/or generation of functional food. According to these results, oxidative stress by Fe (III), at moderate concentrations, could favor the biosynthesis of essential polyunsaturated fatty acids in the early stages of the cultures, probably benefiting from a moderate activity of generation and action of ROS.

### 4.4. Overall

It should be noted here that while the levels of Fe (III) added to the culture medium favor growth (Figure 3), the levels of antioxidant molecules seem to decrease in the case of phenolic compounds. The growth stimulated by Fe (III) is compatible, during the first days of incubation, with a lower whole-cell concentration of chlorophyll (Figure 3b) in cultures with Fe (III). This situation is also compatible with an increase in the efficiency of PSII (Figure 3c), which allows us to postulate that the decrease in the microalgae’s chlorophyll pool might have a regulatory function aimed at maximizing the use of light in grown cultures, as inferred from the photobiochemical data by the increase in ET values and decrease in ABS.

**Figure 8:**
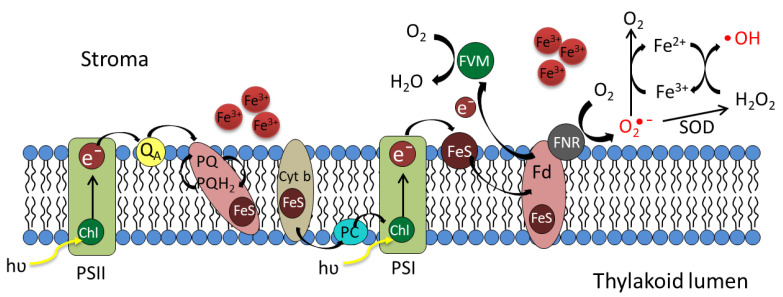
Schematic mechanism proposed for the oxidative stress induction in *C. onubensis*, linked to the photosynthetic chloroplast electron transport chain when Fe (III) is added. PSII: photosystem II; PSI: photosystem I; Chl: chlorophyll; Q_A_: quinone A; PQ: plastoquinone; PQH_2_: plastoquinol; FeS: iron/sulfur cluster belonging to redox proteins; Cyt b: cytochrome b6f; PC: plastocyanin; FVM: like-flavonoid molecules; Fd: ferredoxin; FNR: ferredoxin-NADP^+^ reductase; SOD: superoxide dismutase.

Numerous proteins involved in electron transfer and reductive biosynthetic reactions in microalgae contain or require Fe, as presented in Figure 8. The consequence of that could be an imbalance in the energy that generates through the ETC. The excess energy could react with oxygen to produce ROS through the flavodoxin NADP^+^ reductase (FNR). The generation of hydroxyl radicals through the Fenton reaction could especially cause oxidative damage in functional biomolecules. The result of that is the activation of defense mechanisms such as, e.g., the increase in the SOD enzyme activity (Figure 4a) and the eventual involvement of polyphenols and PUFAs in the antioxidant response of the microalga (Figure 5, Figure 6 and Figure 7). On the other hand, the increase in flavonoids, as mentioned before, could be a mechanism to efficiently release excessive electrons in the ETC (Table 1) by neutralizing oxygen species—without the concomitant production of ROS.

This study could also be consistent with a decrease in the whole-cell content of phenolic compounds due to a lower demand for antioxidant capacity in cultures that grow more efficiently. Simultaneously, the oxidative biochemical consequences of the Fenton reaction in the presence of Fe (III), described in detail in the literature [13], result in the stimulation of the synthesis of antioxidant molecules. Consequently, an increase or decrease in the antioxidant capacity and/or in the whole cell content of certain antioxidant biomolecules in cultures added with Fe (III) should be the result of the balance between the chemical oxidative pressure by ROS against antioxidant biomolecules (ROS being responsible for their apparent decreased content) and the stimulated biosynthesis of antioxidants under the applied stress conditions (responsible for their increased cell levels).

## 5. Conclusions

The addition of moderate concentrations of Fe to cultures of the microalga *C. onubensis* favors the cell growth of the microalga. Fe (III) causes oxidative stress in *Coccomyxa onubensis*. As a defense mechanism, the microalga produces an increase in the intracellular levels of the enzyme superoxide dismutase and flavonoids, while the content of other phenolic molecules suffers from an apparent decrease. The increase in Fe (III) concentrations in the microalgae *C. onubensis* impacts the whole cell content of unsaturated fatty acids; oxidative stress by moderate Fe (III) concentrations seems to favor the biosynthesis of essential polyunsaturated fatty acids, particularly linoleic and linolenic acids, probably as a consequence of a moderate PUFA involvement in ROS neutralization, in addition to a moderate ROS generation activity. Overall, this study offers insight to understand the antioxidant response features of acidophilic/acidotolerant microalgae that can be further useful to design large-scale production of antioxidant molecules-enriched microalgal biomass.

## Figures and Tables

**Table 1:** Photobiochemical parameters were calculated based on the OJIP-polyphases Chl fluorescence signals for the microalga *C. onubensis* as a function of the Fe (III) concentration. Mo, net closing rate of RC during illumination; ABS/RC, absorption flux per RC; TRo/RC, trapping energy flux per RC; ETo/RC, electron transport flux per RC; DIo/RC, dissipated energy flux per RC.

Parameter	Fe (III) Concentration				
	0 mM	0.25 mM	0.5 mM	1 mM	2 mM
Mo	0.210 ± 0.009	0.235 ± 0.011	0.177 ± 0.071 *	0.172 ± 0.007 *	0.164 ± 0.007 *
ABS/RC	1.095 ± 0.047	1.029 ± 0.043 *	1.018 ± 0.046 *	1.034 ± 0.045 *	1.051 ± 0.047 *
TRo/RC	0.807 ± 0.032	0.771 ± 0.034 *	0.766 ± 0.032 *	0.805 ± 0.036	0.795 ± 0.035
ETo/RC	0.597 ± 0.023	0.594 ± 0.025 *	0.603 ± 0.026	0.611 ± 0.027	0.629 ± 0.025
DIo/RC	0.288 ± 0.011	0.257 ± 0.004	0.252 ± 0.005	0.254 ± 0.003	0.256 ± 0.010

(*) Represents the significant differences of all treatments with respect to the control with 95% confidence.

**Table 2:** Maximum average fatty acids content of *C. onubensis* cells incubated in Fe (III)-added culture medium. Fatty acids: palmitic acid (C16:0), hexadecatrienoic acid (C16:3), heptadecenoic acid (C17:1), stearic acid (C18:0), oleic acid (C18:1), α-linoleic acid (C18:2), and α-linolenic acid (C18:3). Data are expressed as a percentage of mg of fatty acid per mg of biomass dry weight. The data exceeding those obtained from control culture samples are highlighted in bold.

Fatty Acid	Fe (III) Concentration				
	0 mM	0.25 mM	0.5 mM	1 mM	2 mM
C16:0	4.35 ± 0.17	4.02 ± 0.17 *	3.18 ± 0.14 *	3.49 ± 0.15 *	3.02 ± 0.12 *
C16:3	0.72 ± 0.04	0.71 ± 0.03	**0.82** ± 0.04 *	0.62 ± 0.02 *	0.65 ± 0.03 *
C17:1	1.10 ± 0.06	**1.55** ± 0.06 *	1.03 ± 0.04 *	**1.12** ± 0.05	0.80 ± 0.03 *
C18:0	1.44 ± 0.04	0.83 ± 0.04 *	0.96 ± 0.04 *	1.11 ± 0.05 *	0.80 ± 0.03 *
C18:1n9	1.08 ± 0.03	**1.10** ± 0.05 *	1.04 ± 0.05 *	0.88 ± 0.04 *	0.88 ± 0.04 *
C18:2n6	2.49 ± 0.11	**2.70** ± 0.12 *	**3.77** ± 0.16 *	2.28 ± 0.09 *	2.30 ± 0.10 *
C18:3n3	3.42 ± 0.15	**4.67** ± 0.20 *	3.17 ± 0.14 *	3.24 ± 0.14 *	2.56 ± 0.12 *

(*) Represents the significant differences of all treatments with respect to the control with 95% confidence.

## Data Availability

All of the data are contained within the article and supplementary materials.

## Supplementary Materials

The following supporting information can be downloaded at: https://www.mdpi.com/article/10.3390/antiox12030610/s1, Figure S1: Time-course evolution of growth in cultures of C. onubensis subjected to different concentrations of Fe (III); Figure S2: Time-course evolution of whole cell chlorophyll concentration in cultures of C. onubensis subjected to different concentrations of Fe (III); Figure S3: Time-course evolution of whole cell chlorophyll a (a) and chlorophyll b (b) concentrations in cultures of C. onubensis subjected to different concentrations of Fe (III).
