# Supplementary material for: Fe (III)-Mediated Antioxidant Response of the Acidotolerant Microalga Coccomyxa onubensis
Source: Antioxidants (Basel). 2023 Mar 1;12(3):610. doi: 10.3390/antiox12030610 (PMC10045799; doi:10.3390/antiox12030610)
Supplement: Supplementary file 1 [file antioxidants-12-00610-s001.zip › antioxidants-2203950-supplementary.pdf]

Fe (III)-Mediated Antioxidant Response of the Acidotolerant Microalga *Coccomyxa onubensis*

María Robles [1], Rafael Torronteras [2], Carol Ostojic [1], Cinta Oria [1], María Cuaresma [1], Inés Garbayo [1], Francisco Navarro [3] and Carlos Vílchez [1,*]

[1] Algal Biotechnology, CIDERTA-RENSMA, Faculty of Experimental Sciences, University of Huelva, 21007 Huelva, Spain
[2] Biology and Environmental Analysis, RENSMA, Faculty of Experimental Sciences, University of Huelva, 21007 Huelva, Spain
[3] Cell Alterations by Exogenous Agents, RENSMA, Faculty of Experimental Sciences, University of Huelva, 21007 Huelva, Spain
* Correspondence: cvilchez@uhu.es; Tel.: +34-959-218-442

Supporting information

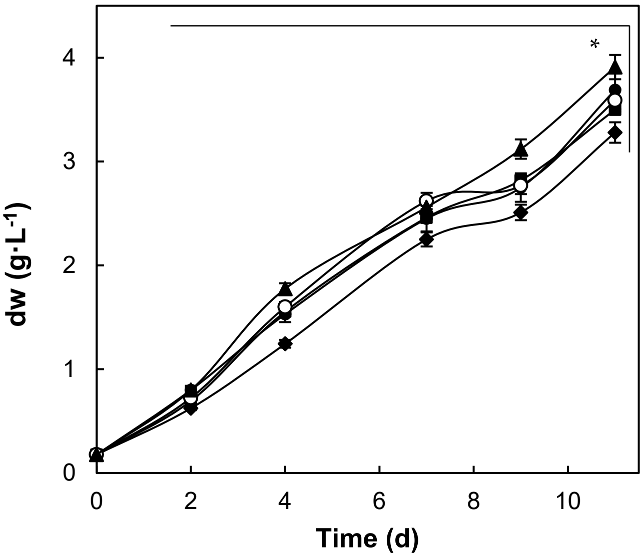

Figure S1. Time-course evolution of growth (dry weight, dw) in cultures of *C. onubensis* subjected to different concentrations of Fe (III). Symbols for Fe (III) concentration: 0 (-◆-), 0.25 (-■-), 0.5 (-●-), 1 (-○-) and 2 (-▲-) mM. The indication Fe (III) 0 mM corresponds to the standard culture medium (control culture). (*) Represents the significant differences of all treatments with respect to the control with 95% confidence.

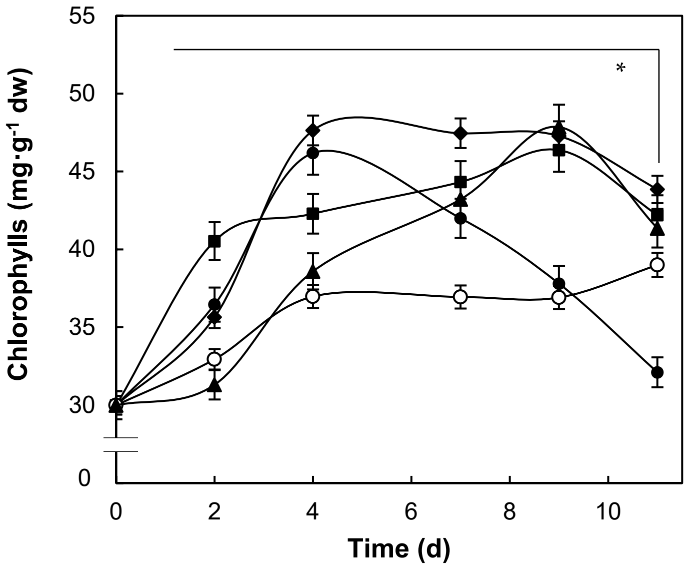

Figure S2. Time-course evolution of intracellular chlorophyll concentration, expressed as mg·g^{-1} dw, in cultures of *C. onubensis* subjected to different concentrations of Fe (III). Symbols for Fe (III) concentration: 0 (-◆-), 0.25 (-■-), 0.5 (-●-), 1 (-○-) and 2 (-▲-) mM. The indication Fe (III) 0 mM corresponds to the standard culture medium (control culture). (*) Represents the significant differences of all treatments with respect to the control with 95% confidence.

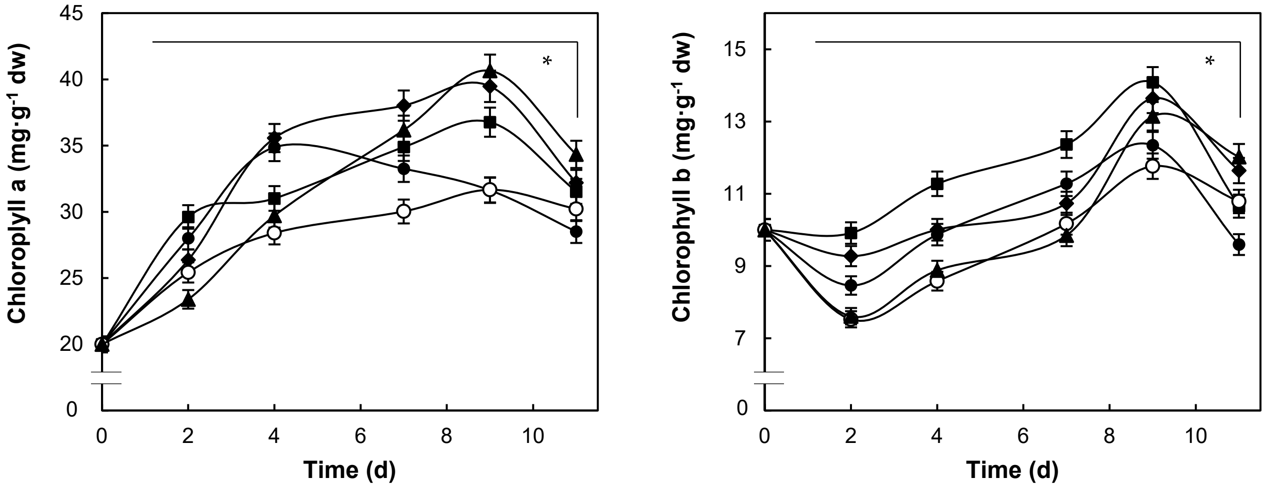

(**a**) (**b**)

Figure S3. Time-course evolution of intracellular chlorophyll a (**a**) and chlorophyll b (**b)** concentrations, expressed as mg·g^{-1} dw, in cultures of *C. onubensis* subjected to different concentrations of Fe (III). Symbols for Fe (III) concentration: 0 (-◆-), 0.25 (-■-), 0.5 (-●-), 1 (-○-) and 2 (-▲-) mM. The indication Fe (III) 0 mM corresponds to the standard culture medium (control culture). (*) Represents the significant differences of all treatments with respect to the control with 95% confidence.
